# Evaluation of seasonal patterns and herd-level traits associated with insemination risk in large dairy herds in Kansas

**DOI:** 10.1371/journal.pone.0217080

**Published:** 2019-05-15

**Authors:** Alexandre L. A. Scanavez, Andréia G. Arruda, Jeffrey S. Stevenson, Luís G. D. Mendonça

**Affiliations:** 1 Department of Animal Sciences and Industry, Kansas State University, Manhattan, Kansas, United States of America; 2 Department of Veterinary Preventive Medicine, The Ohio State University, Columbus, Ohio, United States of America; University of Illinois, UNITED STATES

## Abstract

Adequate identification of estrus is crucial to achieve satisfactory reproductive performance in dairy farms. Even though several studies evaluated expression and identification of estrus at the cow level, limited data exist regarding estrus identification parameters at the herd level. The objectives of this study were to use data from large dairy farms located in Kansas to describe temporal patterns of insemination risk (IR), and to investigate associations between IR and various herd-level factors. Nine herds that housed lactating cows in dry-lots or free-stalls were used in the study. Data from 2012 to 2017 were extracted and categorized in 21-day intervals in a total of 85 cycles, which were classified by season of the year. Mean (SD) IR was 67.6% (4.0) and increased 0.067% (0.009) for each 21-day cycle during the period evaluated. Annual, semi-annual, and trimestral IR peaks were detected using autoregressive integrated moving average analysis. Most of these variations, however, were considered minimal and likely not of economic concern for commercial herds. Insemination risk was greatest during autumn, but did not differ among winter, spring, and summer. Insemination risk was not associated with herd milk yield per season, incidence risk of mastitis during first 21 days in milk, proportion of primiparous cows in the milking herd, or voluntary waiting period of multiparous cows. Herds that housed lactating dairy cows in dry-lots had IR 2.4 percentage points greater than free-stall herds. In addition, mortality during the first 60 days in milk, and category of voluntary waiting period for primiparous cows were associated with IR. In conclusion, seasonal variability in IR was minimal, with increased values observed during the autumn. Insemination risk was greater for dry-lot than free-stall herds. In addition, reduced mortality of lactating cows by 60 days in milk and longer voluntary waiting period for primiparous cows seem to favor greater IR.

## Introduction

Adequate identification of estrus is crucial to achieve satisfactory reproductive performance in dairy farms [1, 2]. Ineffective identification of estrus has a negative impact on insemination risk (**IR**), resulting in extended intervals from parturition to first service [3], increased interval between inseminations [4], and reduced profitability in dairy operations [5]. Despite the recommendation that IR should be greater than 60% for dairy herds [6], considerably poorer results have been reported [2, 7].

Herd IR is influenced by expression and subsequent detection of estrus and can be affected by several factors. In an experiment comparing estrous behavior in two housing systems, Palmer et al. [8] demonstrated that lactating cows housed in free-stall facilities express less evident signs of estrus than cows managed on pasture. In addition, Vailes and Britt [9] suggested that footing surface can impact expression of estrus. Shorter duration and fewer mounting events during estrus also are associated with high milk yield [10] and lameness [11]. Furthermore, expression of estrus is suppressed during periods of heat stress [12–15], which could lead to poor estrus-detection risk [16] and IR.

In many herds, daily observation for signs of estrus is improperly performed [16]. In order to facilitate this task and improve accuracy of detection, various strategies have been adopted [4], such as pressure-sensitive patches [17], pedometers [18], and activity monitoring systems [19]. Although growing interest in adoption of these technologies has occurred, many large dairies in the U.S. rely on applying paint or chalk on the tail head of cows for detection of mounts received [16]. In a recent study conducted by our research group with heat-stressed cows [20], more than 89% of first inseminations were based on tail paint removal, which occurred between 53 and 84 days in milk. Therefore, it is plausible to infer that greater IR may be achieved in dairies with efficient reproductive management that rely largely on tail paint removal for insemination, regardless of season of the year.

Several studies evaluated expression and identification of estrus at the cow level [8, 21, 22], but limited data exist regarding estrus identification parameters at the herd level (e.g., IR). It is unclear whether reduction of expressed estrus during the warm season of the year compromises IR at the herd level. A recent report [23] presented evidence that reduction in IR during the summer is subtle in large dairies. In this study, annual average IR for 25 herds was 66% in 2015, and during the summer IR was only 2.6 percentage units worse than IR achieved for the remainder of the year. Therefore, it is possible that reduced estrual behavior of dairy cows during summer can be compensated by management practices such as performing tail painting to increase estrous detection, resulting in modest variations in IR across the year. In addition, it is not clear how housing systems for lactating dairy cows influence herd-level IR across different seasons. Because herds in the Great Plains of Kansas have a variety of housing systems, excellent reproductive performance (e.g., annual 21-day pregnancy risk greater than 22% [6, 23]), and cows are subjected to heat stress conditions [23, 24], this region and cohort of herds present ideal conditions for studying herd-level aspects related to reproductive success across seasons.

The objectives of this study were to use data from large dairy farms located in Kansas to describe temporal patterns of IR, and to investigate the association between IR and several factors, including housing, milk yield, voluntary waiting period (**VWP**), proportion of primiparous cows in the milking herd, mastitis incidence, and mortality. We hypothesized that IR would be: (1) without seasonal peaks or nadirs; (2) greater in dry-lot facilities compared with free-stall herds; and (3) negatively associated with milk yield, mastitis incidence, mortality, and proportion of primiparous cows in the milking herd, and positively associated with VWP.

## Materials and methods

### Inclusion criteria

Dairy herds located in Kansas (n = 9) with a minimum of 1,500 lactating cows, with at least 6 years of records and that were enrolled in the Kansas State University extension program to monitor herd performance were eligible to be enrolled in the study. Only herds that achieved IR ≥ 60% during 2017 were included in the study population. Among these herds, housing systems for lactating cows were either dry-lot corrals (n = 5) or free-stall facilities with access to dirt exercise lots (n = 4). All herds used artificial insemination (**AI**) as the exclusive method of breeding, and recorded information daily in on-farm management software (Dairy Comp 305, Valley Ag Software, Tulare, CA). Differences observed in reproductive management among herds included frequency of pregnancy diagnosis, VWP, days in milk at which non-inseminated cows were submitted to a timed AI program for first service, and synchronization programs. Although reproductive programs were dissimilar among herds, the majority of inseminations were performed based on tail paint removal in all herds. A small proportion of inseminations were coded as timed AI in the management software, which ranged from 3 to 32% with an overall average of 13% across herds. Minor changes occurred in reproductive management of each herd during the period analyzed in this study. These changes included alterations in VWP and, to a lesser extent, synchronization programs.

The researchers did not apply treatments or have contact with animals during any phase of this study. All data used were extracted from a database of records from dairy herds. Therefore, approval from an animal research ethics committee was not required for completion of this observational study.

### Variables of interest and data collection

Insemination risk was the outcome of interest and was calculated in Microsoft Excel (Microsoft Corp., Redmond, WA) using the following formula: IR = number of cows inseminated during a 21-day period divided by the number of cows eligible to be inseminated during the 21-day period. Values for the calculation of IR were extracted from the on-farm management software. Cows were considered eligible to AI based on: (1) VWP of primiparous and multiparous within each herd; (2) non-pregnancy status; (3) not elected to be culled at the end of lactation; and (4) remaining in the herd for at least 11 days of the specific 21-day cycle being analyzed. All inseminations performed in a given 21-day cycle were considered for IR calculation, regardless of being performed based on estrous detection or timed AI. Three herds practiced different VWP for primiparous (lactation = 1) and multiparous (lactation > 1) cows. Therefore, VWP was adjusted by parity within herd. Data from December 2012 to November 2017 were extracted and 21-day cycles were numbered from 1 to 17 for each year. Within each year, the first 21-day cycle was calculated starting on December 1^st^, and the 17^th^ cycle starting on November 2^nd^. Cycles were categorized by season of the year based on the majority of days in a cycle occurring in a given season: winter (cycles 1 through 4); spring (cycles 5 through 9); summer (cycles 10 through 13); or autumn (cycles 14 through 17).

Explanatory variables explored were housing systems (dry-lot vs. free-stall), VWP (in days) of primiparous (**VWP-P**) and multiparous (**VWP-M**) cows, season of the year (winter, spring, summer, and autumn), proportion of primiparous cows in the milking herd (**% primiparous**), herd milk yield per season (in kg), and herd incidence risk of mastitis during the first 21 days in milk (**% mastitis**) and mortality during the first 60 days in milk (**% dead**). Proportions of primiparous cows in the milking herd and incidence risks of mastitis and mortality also were extracted in a 21-day timeframe to coincide with the 21-day cycles previously described (cycles numbered 1 to 17). For regression analyses, weighted averages of the variables were calculated for each season. Milk yield was recorded monthly at each dairy, and weighted averages were calculated for each season. Voluntary waiting periods of primiparous and multiparous cows were evaluated for each season and were categorized as: < 50, 50 to 54, 55 to 59, or ≤ 60 days in milk.

### Statistical analyses

#### Descriptive data

Continuous data were screened for normality with histograms using Stata/IC 15.1 (StataCorp LLC, College Station, TX). Mean (SD) number of cows, VWP-P, VWP-M, % primiparous, milk yield, % mastitis, and % dead during the period of the study were calculated for each herd. In addition, descriptive data stratified by production system (e.g., dry-lot or free-stall) were also explored.

#### Time-series analysis

Cycles of 21 days were selected as the measurement unit for analysis of trends and seasonal effects of IR because this interval is widely adopted in the dairy industry for monitoring IR and overall reproductive efficiency. Insemination risk in each cycle was the outcome of interest. Data from all herds were combined, and a total of 85 cycles between December 2012 and November 2017 were available for analyses. Trends and seasonal effects on IR were assessed using autoregressive integrated moving averages (**ARIMA**), as previously described [25, 26]. Raw and decomposed IR by cycle data were plotted to allow for visual inspection of trends and seasonality. Autocorrelation (**ACF**) and partial autocorrelation plots were generated to assess correlations among residuals. Using ARIMA modeling with temporal trends as covariates, presence of annual, semiannual, and trimestral seasonality was tested using multivariable linear regressions models. Variables with p*-*value > 0.10 were excluded from the base model by backwards elimination. The final ARIMA model was selected based on lowest Akaike Information Criterion (**AIC**), and was assessed with the Box-Pierce test. To ensure the final model resulted in a stationary series, residuals correlations were again screened using ACF and partial ACF. Analyses were conducted using the ‘forecast’ package [27] of RStudio 1.0.44 (RStudio, Boston, MA).

#### Regression model

Data used for the regression analyses were collected at the herd level. A causal diagram containing the outcome (IR), the main explanatory variable of interest (housing type), and other variables investigated was drawn to aid identification of possible confounders. Linearity between continuous predictors and IR was visually inspected using the lowess procedure of Stata, and statistically assessed using the reg procedure. Presence of multicollinearity among study variables was evaluated using the Spearman correlation coefficient. Two variables were considered correlated when the coefficient of correlation was > 0.8, which did not happen for any of the combinations of variables tested. Univariable linear regression analyses using IR as dependent variables were conducted for each predictor using dairy as random effect in Stata. Variables with p*-*value > 0.20 were deemed not significant and were not eligible to be included in the base model, unless they were identified as confounders on the causal diagram.

Remaining variables were included in the base model using dairy as a random effect, and backwards elimination was used to exclude nonsignificant variables. Statistical significance was defined as p*-*value ≤ 0.05 and tendencies as 0.05 < p ≤ 0.10. Distribution of best linear unbiased residuals was assessed to evaluate the final model’s fit by plotting them in Stata and visually inspecting for normality.

## Results

Mean (SD) number of cows and IR were 4,578 (2,028) and 67.6% (4.0), respectively. Further descriptive data stratified by housing type are presented on Table 1.

**Table 1:** Descriptive data for the nine large Kansas dairy herds enrolled in this study. Data are presented as mean (SD, minimum, maximum).

Housing type	Number of lactating cows	Insemination risk (%)	VWP-P^1^ (days)	VWP-M^2^ (days)	% primiparous^3^	Milk yield (kg/cow/day)	**% mastitis**^4^	% dead^5^
Dry-lot	4,633 (2,637, 1,596, 9,562)	68.8 (4.2, 55.7, 74.6)	53.9 (6.5, 45.0, 65.0)	50.3 (2.5, 48.0, 58.0)	40.1 (3.0, 30.5, 46.3)	32.6 (2.1, 28.4, 36.9)	3.8 (2.5, 0.3, 14.7)	3.2 (1.7, 0.7, 8.2)
Free-stall	4,509 (773, 2,755, 5,693)	65.9 (3.3, 57.9, 75.7)	53.1 (4.0, 50.0, 65.0)	52.9 (3.7, 50.0, 65.0)	42.6 (3.5, 35.9, 49.9)	34.6 (2.2, 29.8, 39.9)	4.8 (2.6, 1.4, 15.7)	3.0 (1.2, 1.0, 7.0)
All herds	4,578 (2,028, 1,596, 9,562)	67.6 (4.0, 55.7, 75.7)	53.6 (5.5, 45.0, 65.0)	51.4 (3.3, 48.0, 65.0)	41.2 (3.4, 30.5, 49.9)	33.5 (2.3, 28.4, 39.9)	4.3 (2.6, 0.3, 15.7)	3.1 (1,5, 0.7, 8.2)

^1^ Voluntary waiting period of primiparous cows

^2^ Voluntary waiting period of multiparous cows

^3^ Proportion of primiparous cows in the lactating herd

^4^ Herd incidence risk of mastitis within 21 days in milk

^5^ Mortality of lactating dairy cows during the first 60 days in milk

**Fig 1:**
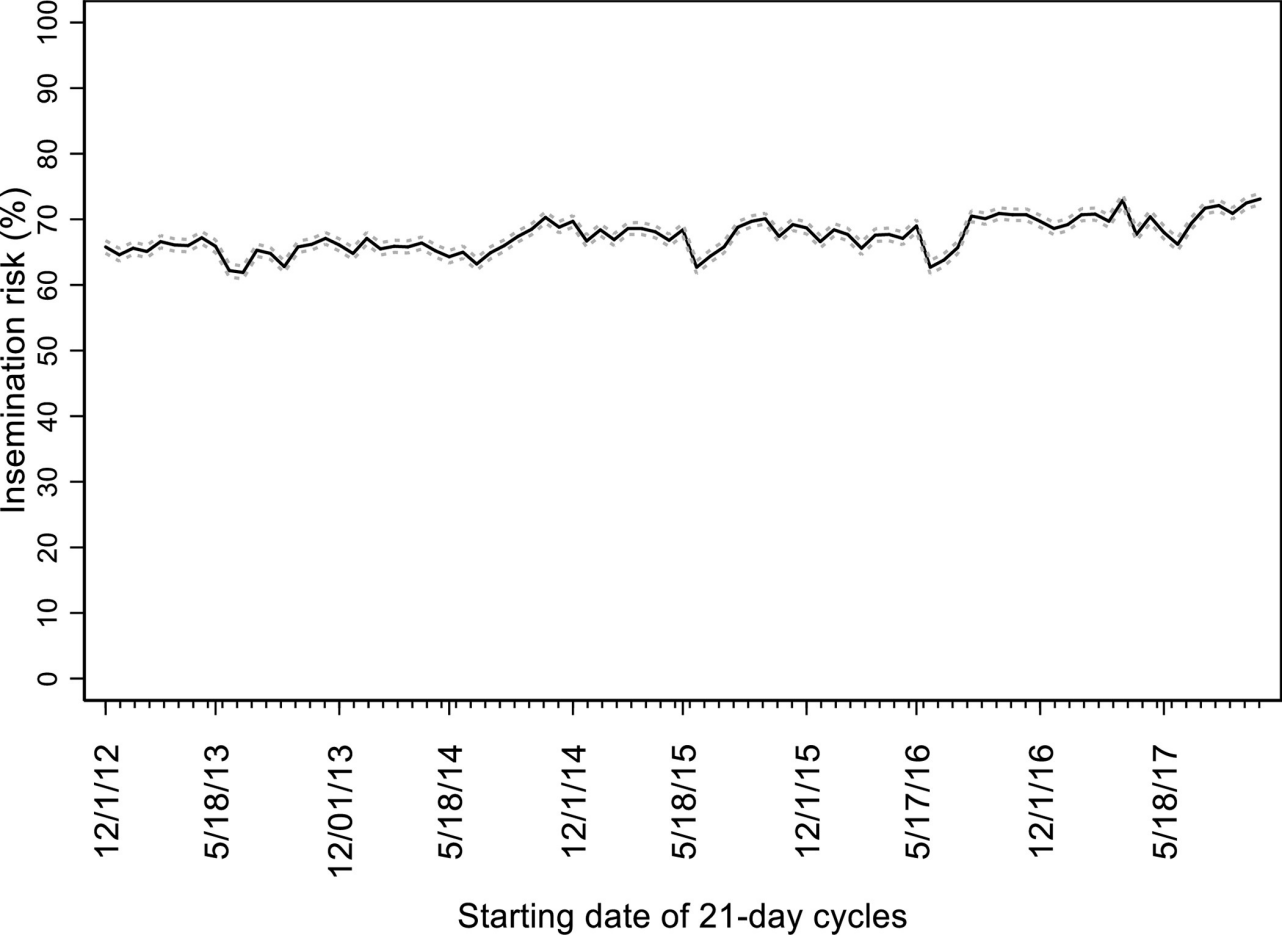
Insemination risk of 9 large dairy herds located in Kansas. Black solid line represents mean insemination risk, and gray dotted lines represent 95% confidence interval for 21-day cycles (n = 85) starting December 1, 2012 through November 2, 2017.

**Fig 2:**
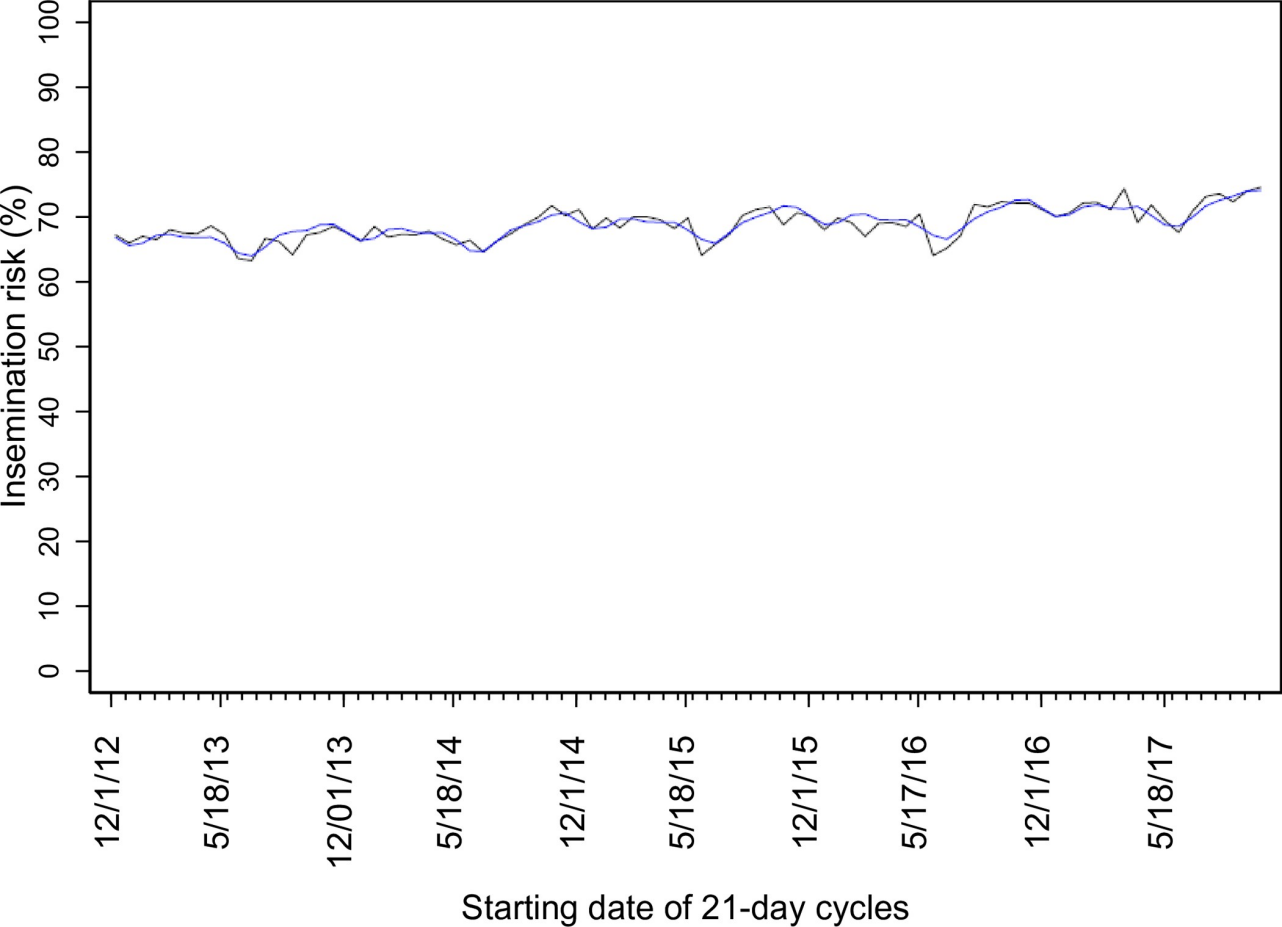
Time series model fit. Black and blue lines represent actual and estimated values for insemination risk, respectively.

Mean IR with a 95% confidence interval for each 21-day cycle for the herds is depicted in Fig 1. Insemination risk increased (p < 0.01) on average (SE) 0.067% (0.009) for each 21-day cycle in the time series analyzed. In addition, annual, semi-annual, and trimestral IR peaks were detected (Table 2). The final ARIMA model showed an overall good fit, with p-value = 0.83 in the Box-Pierce test. Model fit is shown in Fig 2.

**Table 2:** Final autoregressive integrated moving average (ARIMA) model^1^ used to evaluate presence of seasonal peaks in insemination risk in large dairy herds located in Kansas.

Item	Fixed effect estimate	SE	p*-*value
Intercept	64.56	0.45	
Overall trend	0.07	0.01	< 0.01
Annual peaks	1.22 cos (2πt/17)	0.21	< 0.01
	0.58 sin (2πt/17)	0.22	< 0.01
Semi-annual peaks	0.31 cos (2πt/8.5)	0.19	< 0.01
	-1.27 sin (2πt/8.5)	0.19	0.11
Trimestral peaks	0.62 cos (2πt/4.25)	0.18	0.96
	0.01 sin (2πt/4.25)	0.18	< 0.01
Y_t_	0.84Y_t-1_	0.13	
	Zt—0.72 Z_t-1_	0.15	

^1^ ARIMA type = (1,0,1). AIC = 301.3

The final multivariable mixed effect regression model showed that milk yield, % mastitis, % primiparous, and VWP-M were not associated (p < 0.16) with IR. The variables retained in the final multivariable regression model were housing system, season of the year, % dead, and VWP-P. Lactating dairy cows housed in dry-lots had greater (p > 0.01) IR than free-stall herds (Table 3). Insemination risk was associated with season of the year because IR was greatest during autumn. Nonetheless, IR did not differ among winter, spring, and summer (p > 0.16). In addition, % dead and VWP-P were associated (p < 0.01) with IR (Table 3).

**Table 3:** Final multivariable linear regression model used to evaluate the association of various herd-level factors and insemination risk in large dairy herds located in Kansas.

Item	Fixed effect estimate (SE)	95% CI^3^ for fixed effect estimate	p*-*value
Intercept	72.4 (1.2)	70.0 to 74.8	
Housing system			
Dry-lot	Referent^a^		
Free-stall	-2.4 (0.8)^b^	-3.9 to -0.90	< 0.01
Season of the year			
Autumn	Referent^a^		
Winter	-1.79 (0.5)^b^	-2.9 to -0.7	< 0.01
Spring	-1.7 (0.5)^b^	-2.8 to -0.7	< 0.01
Summer	-2.5 (0.6)^b^	-3.6 to -1.4	< 0.01
% dead^1^	-1.1 (0.2)	-1.5 to -0.8	< 0.01
VWP- P category^2^			
45	Referent^ab^		
50	1.0 (1.2)^a^	-1.3 to 3.4	0.38
55	-0.7 (1.3)^b^	-3.2 to 1.9	0.61
60	4.0 (1.2)^c^	1.6 to 6.4	< 0.01

^1^ Mortality of lactating dairy cows from parturition until 60 days in milk

^2^ Category of voluntary waiting period for primiparous cows: 45 = 45 to 49 days in milk; 50 = 50 to 54; 55 = 55 to 59; 60 = 60 to 65

^3^ Confidence interval

^a,b,c^ Values within a column-item with unlike superscript letters differ (p-value < 0.05)

## Discussion

Insemination risk is a key performance indicator that directly affects pregnancy risk and has a significant impact on profitability of dairy herds [5]. Because previous peer-reviewed literature indicates that estrous expression is reduced during periods of heat stress [13–15], IR is anecdotally considered to be seasonal, despite the lack of herd-level investigations to support this speculation. Recent findings [23] indicate that seasonal variation in IR might be minor in large dairy herds achieving IR > 60%. Nonetheless, time series analysis was not conducted in the previous report [23], limiting major inferences. Therefore, the present study was conducted to evaluate the existence of seasonal patterns of IR in large dairy herds by using time series analysis, and to investigate other herd-level factors associated with IR.

In the current study, overall IR for the enrolled herds showed an increasing trend during the 5-year period analyzed (2012 through 2017). Previous reports demonstrated contrasting trends, with IR reducing over time [7]. The latter study, however, was conducted using data collected between 1985 and 1999, when specific practices and technologies, such as tail painting for estrous detection or timed AI, were not yet widely employed. Substantial changes have recently occurred in the dairy industry with implementation of modern management practices focusing on improving animal health, welfare, and fertility, which likely contributed to improvements in IR observed during recent years [23]. Contrary to our initial hypothesis, our data revealed seasonal patterns of IR, which was consistent during winter, spring, and summer, but increased during autumn. Even though the time series analysis suggested annual, semi-annual, and trimestral peaks, most of these variations seem to be minimal and likely not meaningful for commercial herds. We speculate that the increased IR during autumn might have occurred as a consequence of poor fertility commonly observed during summer [28], which results in more cows eligible to be inseminated during autumn. In addition, a large proportion of calvings commonly occur during summer months in dairy herds affected by heat stress [29], resulting in an increased number of cows submitted to first AI during autumn. Therefore, it is possible that IR is increased after summer because of more pronounced sexual behavior resulting from a greater proportion of cows in estrus during the same period [30], facilitating estrous detection and increasing IR. The IR difference between summer and autumn was not nearly as large as the decrease in estrous expression previously reported in cows under heat stress [12, 31]. Therefore, we speculate that the reduced estrous expression observed at the cow level during summer months might be compensated by management practices such as daily observation of cows for signs of estrus or use of tail painting, resulting in minimal disturbance to herd-level indicators of estrous expression, such as IR.

In addition to exploring occurrence of seasonal trends, the present study was designed to investigate the relationship between herd-level characteristics and IR. As initially hypothesized, IR was greater for dry-lot compared with free-stall herds. Vailes and Britt [9] demonstrated that mounting activity was 3 to 15 times greater when cows were in estrus on a dirt surface compared with cows housed on concrete. Vailes and Britt [9], however, used estrogen-treated ovariectomized cows, and a small sample size, which prevents extrapolation of conclusions to commercial settings. Furthermore, Palmer et al. [8] concluded that estrous detection using visual observation, tail painting, or radiotelemetry were less effective when cows were in free-stall facilities compared with cows in pasture paddocks. According to the latter study, such difference was observed because standing behavior was reduced in cows managed in the free-stall barns. The difference in IR between free-stall and dry-lot herds was minor in our study (−2.4 ± 0.8%). We speculate that the reduced expression of estrous behavior in free-stall herds was partially compensated by efficient estrus-detection programs, and consequently, only subtle differences were observed among cows in different housing types.

Health during the first weeks of lactation is an important determinant of reproductive success in dairy operations [32]. In the current study, however, % mastitis was not associated with herd-level IR. In research projects conducted with grazing [33] and confined dairy cows [34], researchers concluded that cows presenting mastitis during the first postpartum weeks did not present delayed resumption of luteal activity, which supports our findings. It is important to note that others [33, 34] focused on cow-level outcomes instead of the herd-level approach used in the current study. Nevertheless, these studies clearly demonstrate the deleterious effects of other postpartum health disorders on resumption of luteal activity [33, 34], which can potentially affect IR. Another interesting finding from the current study was the negative association between % dead and IR. Considering that health status of early lactating cows likely influences % dead, it is possible that an increased percentage of cows with health disorders had negative implications on estrous expression [35], and ultimately, affected IR. A recent report [36] indicated that % dead is associated with pregnancy per AI of multiparous cows in large commercial herds. Nevertheless, to the best of our knowledge, studies focusing on the relationship between mortality during the first 60 days after calving and IR are still lacking.

Proportion of primiparous cows (% primiparous) in the lactating herd was not associated with IR of the herds used in the study. This finding is intriguing considering that primiparous cows are expected to have delayed resumption of luteal activity. Primiparous cows have greater postpartum concentrations of non-esterified fatty acids in plasma than multiparous cows [37]. Increased concentration of non-esterified fatty acids is linked to negative energy balance, which is associated with delayed ovarian function (anovulation) and results in primiparous cows being more likely to be anovulatory at the end of the VWP than multiparous cows [38]. Nonetheless, an increased proportion of primiparous cows did not have a reduced IR. We speculate that negative energy balance was not excessive in these herds and prevented % primiparous cows from contributing negatively to IR. Because a positive relationship exists between proportion of cows resuming luteal activity and days postpartum, VWP duration was explored as a risk factor for decreased IR. In the current study, VWP was positively associated with IR for primiparous, but not for multiparous cows. Periparturient primiparous cows are more likely to develop metritis than multiparous cows [39], which is a risk factor for cows to not resume luteal activity [33, 34]. Findings from the current study corroborate with results reported by Santos et al. [38], who demonstrated that a smaller proportion of primiparous than multiparous cows resumed luteal activity by 65 days in milk. Altogether, it is likely that extending VWP for primiparous cows favors IR. In addition, use of ovulation-synchronization programs for first AI for all primiparous cows enrolled in programs with extended VWP may have caused a greater proportion of cows to be inseminated for the first time shortly after the end of the VWP, also increasing IR.

No association was detected between milk yield and IR in the study population. In a study conducted with cows with high milk yield, reduced number and shorter duration of standing events during estrus were reported [10]. Reduced intensity of sexual behavior may theoretically pose a challenge for estrous detection, given the reliance on tail paint removal in the U.S. to detect cows in estrus [16]. The number of mounts or total duration of mounting events to trigger estrous detection aids (e.g., tail paint removal) has not been quantified and would depend on the product used. Therefore, it is possible that the negative association of increased milk yield on estrous behavior is not sufficient to decrease estrus-detection risk in herds similar to the ones enrolled in this study, resulting in satisfactory IR. Nonetheless, it is important to consider that average milk yield of the herd was used in the analyses of the current study, which differs from other reports that used a cow-level approach to study the association between productivity and estrus expression [10, 40]. It is important to acknowledge that milk yield of the herds used in this study is lower than previously reported in the literature [10, 22]. It is possible that an association between IR and herd-level milk yield exists in herds with increased milk production, which should be investigated in future studies.

The authors recognize that inclusion of herds located in various geographic areas would have increased the external validity of the present study. It is important to note, however, that approximately 42% of the herds with more than 500 cows in the U.S. house lactating cows in similar facilities as the herds used in this study [41]. In addition, this cohort of herds is projected to grow considerably faster than smaller herds in the future [41], which should augment the external validity of our findings. We also recognize the possibility of misclassification of mastitis cases, given that diagnoses were performed by farm personnel and clinical definition could vary slightly among herds. To the best of our knowledge, this is the first peer-reviewed report to demonstrate that various herd-level characteristics are associated with IR. In addition, findings of the current study show the inverse relationship between IR and postpartum mortality of lactating cows, which indicates the benefit of optimizing overall herd health to have a successful reproductive program.

In conclusion, seasonal variability in IR was minimal in the population studied and increased values were observed during the autumn. Insemination risk was greater for dry-lot than free-stall herds. In addition, reduced mortality of lactating cows by 60 days in milk and longer VWP for primiparous cows were positively associated with IR. Further research is warranted to evaluate the association of other factors with IR and to validate our findings in other geographic areas and production systems.

## References

[1] Senger PL. The estrus detection problem: new concepts, technologies, and possibilities. J Dairy Sci. 1994; 77:2745–2753. 10.3168/jds.S0022-0302(94)77217-9 7814743

[2] Kinsel ML, Etherington WG. Factors affecting reproductive performance in Ontario dairy herds. Theriogenology. 1998; 50:1221–1238. 10734437 10.1016/s0093-691x(98)00222-2

[3] Stevenson JS, Call EP. Influence of early estrus, ovulation, and insemination on fertility in postpartum Holstein cows. Theriogenology. 1983; 19:367–375.

[4] Fricke PM, Carvalho PD, Giordano JO, Valenza A, Lopes G Jr, Amundson MC. Expression and detection of estrus in dairy cows: the role of new technologies. Animal. 2014; 8:134–143. 10.1017/S1751731114000299 24680286

[5] Cabrera VE. Economics of fertility in high-yielding dairy cows on confined TMR systems. Animal. 2014; 8:211–221. 10.1017/S1751731114000512 24679357

[6] Mendonça LGD. Metrics to assess reproductive efficiency in dairy herds. Proceedings of the 10th Dairy Cattle Reproduction Council conference; 2015 Nov 11–13; Buffalo, NY. Dairy Cattle Reproduction Council; 2015. p. 83–93.

[7] Washburn SP, Silvia WJ, Brown CH, McDaniel BT, McAllister AJ. Trends in reproductive performance in southeastern Holstein and Jersey DHI herds. J Dairy Sci. 2002; 85:244–251. 10.3168/jds.S0022-0302(02)74073-3 11860117

[8] Palmer MA, Olmos G, Boyle LA, Mee JF. Estrus detection and estrus characteristics in housed and pastured Holstein-Friesian cows. Theriogenology. 2010; 74:255–264. 10.1016/j.theriogenology.2010.02.009 20451993

[9] Vailes LD, Britt JH. Influence of footing surface on mounting and other sexual behaviors of estrual Holstein cows. J Anim Sci. 1990; 68:2333–2339. 2401655 10.2527/1990.6882333x

[10] Lopez H, Satter LD, Wiltbank MC. Relationship between level of milk production and estrous behavior of lactating dairy cows. Anim Reprod Sci. 2004; 81:209–223. 10.1016/j.anireprosci.2003.10.009 14998648

[11] Sood P, Nanda AS. Effect of lameness on estrous behavior in crossbred. Theriogenology. 2006; 66:1375–1380. 10.1016/j.theriogenology.2006.04.031 16765429

[12] Pennington JA, Albright JL, Diekman MA, Callahan CJ. Sexual activity of Holstein cows: seasonal effects. J Dairy Sci. 1985; 68:3023–3030. 4078128 10.3168/jds.S0022-0302(85)81197-8

[13] Younas M, Fuquay JW, Smith AE, Moore AB. Estrus and endocrine responses of lactating Holsteins to forced ventilation during summer. J Dairy Sci. 1993; 76:430–434. 10.3168/jds.S0022-0302(93)77363-4 8445097

[14] Cartmill JA, El-Zarkouny SZ, Hensley BA, Rozell TG, Smith JF, Stevenson JS. An alternative AI breeding protocol for dairy cows exposed to elevated ambient temperatures before or after calving or both. J Dairy Sci. 2001; 84:799–806. 10.3168/jds.S0022-0302(01)74536-5 11352155

[15] De Rensis F, Scaramuzzi R J. Heat stress and seasonal effects on reproduction in the dairy cow: a review. Theriogenology. 2003; 60:1139–1151. 12935853 10.1016/s0093-691x(03)00126-2

[16] Caraviello DZ, Weigel KA, Fricke PM, Wiltbank MC, Florent MJ, Cook NB, et al Survey of management practices on reproductive performance of dairy cattle on large US commercial farms. J Dairy Sci. 2006; 89:4723–4735. 10.3168/jds.S0022-0302(06)72522-X 17106104

[17] Badinga L, Collier RJ, Thatcher WW, Wilcox CJ. Effects of climatic and management factors on conception rate of dairy cattle in subtropical environment. J. Dairy Sci. 1985; 68:78–85. 10.3168/jds.S0022-0302(85)80800-6 3980812

[18] Lehrer AR, Lewis GS, Aizinbud E. Oestrus detection in cattle: recent developments. Anim Reprod Sci. 1992; 28:355–361.

[19] Jónsson R, Blanke M, Poulsen NK, Caponetti F and Højsgaard S. Oestrus detection in dairy cows from activity and lying data using on-line individual models. Computers and Electronics in Agriculture. 2011; 76:6–15.

[20] Voelz BE, Rocha L, Scortegagna F, Stevenson JS, Mendonça LGD. Treatment of lactating dairy cows with gonadotropin-releasing hormone before first insemination during summer heat stress. J Dairy Sci. 2016; 99:7612–7623. 10.3168/jds.2016-10970 27289155

[21] Xu ZZ, Knight DJ, Vishwanath R, Pitt CJ, Burton LJ. Estrus detection using radio telemetry or visual observation and tail painting for dairy cows on pasture. J Dairy Sci. 1998; 81:2890–2896. 10.3168/jds.S0022-0302(98)75849-7 9839231

[22] Valenza A, Giordano JO, Lopes G Jr., Vincenti L, Amundson MC, Fricke PM. Assessment of an accelerometer system for detection of estrus and treatment with gonadadotropin-releasing hormone at the time of insemination in lactating dairy cows. J Dairy Sci. 2012; 95:7115–7127. 10.3168/jds.2012-5639 23040033

[23] Scanavez A, Voelz BE, Mendonça L. Benchmarking reproductive efficiency and transition cow health of Kansas dairy herds. Kansas Agricultural Experiment Station Research Reports. 2016 Vol. 2.

[24] Voelz B E, Payne C E, Hulbert L, Stevenson J S, Brouk M, Mendonça L G D. Kansas dairy producers' needs survey: reproductive management on Kansas dairy farms. J Ext. 2017; 55 (4), article # 4RIB6.

[25] Alba A, Dórea FC, Arinero L, Sanchez J, Cordón R, Puig P, et al Exploring the Surveillance Potential of Mortality Data: Nine Years of Bovine Fallen Stock Data Collected in Catalonia (Spain). PLOS ONE. 2015 4 15; 10(4):e0122547 10.1371/journal.pone.0122547 25876036 PMC4398401

[26] Arruda AG, Vilalta C, Puig P, Perez A, Alba A. Time-series analysis for porcine reproductive and respiratory syndrome in the United States. PLOS ONE. 4 3; 13(4): e0195282 10.1371/journal.pone.0195282 29614099 PMC5882168

[27] Hyndman R J, Khandakar Y. Automatic time series forecasting: the forecast package for R. J Stat Softw. 2008 7; 27 (3).

[28] Jordan ER. Effects of heat stress on reproduction. J Dairy Sci. 2003: (E. Suppl.):E104– E114.

[29] Mendonça LGD, Mantelo FM, Stevenson JS. Fertility of lactating dairy cows treated with gonadotropin-releasing hormone at AI, 5 days after AI, or both, during summer heat stress. Theriogenology. 2017; 91:9–16. 10.1016/j.theriogenology.2016.11.032 28215691

[30] Hurnik JF GJ King, Robertson HA. Estrus and related behaviour in postpartum Holstein cows. Applied Animal Ethology. 1975; 2:55–68.

[31] Schüller LK, Michaelis I, Heuwieser W. Impact of heat stress on estrus expression and follicle size in estrus under field conditions in dairy cows. Theriogenology. 2017; 102:48–53. 10.1016/j.theriogenology.2017.07.004 28743027

[32] Santos JEP, Ribeiro ES. Impact of animal health on reproduction of dairy cows. Anim Reprod. 2014; 11:254–269.

[33] Ribeiro ES, Lima FS, Greco LF, Bisinotto RS, Monteiro APA, Favoreto M, et al Prevalence of periparturient diseases and impacts on fertility of seasonally calving grazing dairy cows supplemented with concentrates. J Dairy Sci. 2013; 96:5682–5697. 10.3168/jds.2012-6335 23831093

[34] Santos JEP, Bisinotto RS, Ribeiro ES, Lima FS, Greco LF, Staples CR, at al. Applying nutrition and physiology to improve reproduction in dairy cattle. Soc Reprod Fertil. 2010; 67:387–403. 10.7313/upo9781907284991.030 21755686

[35] Rutherford AJ, Oikonomou G, Smith RF. The effect of subclinical ketosis on activity at estrus and reproductive performance in dairy cattle. J Dairy Sci. 2016; 99:4808–4815. 10.3168/jds.2015-10154 26995121

[36] Mendonça L, Scanavez A. Association between reproduction and postpartum cow health during summer months in dairies located in the Great Plains Region. Kansas Agricultural Experiment Station Research Reports: 2017; Vol. 3.

[37] Wathes DC, Cheng Z, Bourne N, Taylor VJ, Coffey MP, Brotherstone S. Differences between primiparous and multiparous dairy cows in the inter-relationships between metabolic traits, milk yield and body condition score in the periparturient period. Domest Anim Endocrinol. 2007; 33: 203–225. 10.1016/j.domaniend.2006.05.004 16806790

[38] Santos JEP, Rutigliano HM, Sá Filho MF. Risk factors for resumption of postpartum estrous cycles and embryonic survival in lactating dairy cows. Anim Reprod Sci. 2009; 110:207–221. 10.1016/j.anireprosci.2008.01.014 18295986

[39] Goshen T, Shpigel NY. Evaluation of intrauterine antibiotic treatment of clinical metritis and retained fetal membranes in dairy cows. Theriogenology. 2006; 66:2210–2218. 10.1016/j.theriogenology.2006.07.017 16962164

[40] Rivera F, Narciso C, Oliveira R, Cerri RLA, Correa-Calderon A, Chebel RC, et al Effect of bovine somatotropin (500 mg) administered at ten-day intervals on ovulatory responses, expression of estrus, and fertility in dairy cows. J Dairy Sci. 2010; 93:1500–1510. 10.3168/jds.2009-2489 20338427

[41] USDA. 2016. Dairy 2014, dairy cattle management practices in the United States. https://www.aphis.usda.gov/animalhealth/nahms/dairy/downloads/dairy14/Dairy14drPartI.pdf

